# Early Treatment and Functional Recovery in Treatment-Resistant Depression: Real-World Evidence from Intranasal Esketamine in Spain

**DOI:** 10.1192/j.eurpsy.2026.10613

**Published:** 2026-07-31

**Authors:** A. I. De Santiago-Diaz, M. A. Madrazo-Maza, C. Pavón-Navajas, A. Pérez-Poza, J. E. Mesones-Peral, E. Sáez-Fuentes, F. Mora-Mínguez, M. Arrojo-Romero, A. Miranda-Sivelo, F. A. Rodríguez-Batista, V. Roselló-Molina, L. Gutiérrez-Rojas, V. Tordera-Tordera, I. Domingos-Chaves, D. Núñez-Arias, L. Montes Reula, S. Arostegui-Uranga

**Affiliations:** 1 Psychiatry, University Hospital Valdecilla, Santander; 2 University Hospital Basurto, Bilbao; 3 Psychiatric Hospital of Araba, Vitoria; 4 Psychiatry, University Hospital Miguel Servet, Zaragoza; 5 Psychiatry, University Hospital of Torrevieja, Torrevieja; 6 Psychiatry, Hospital of Galdákano, Vizcaya; 7 Psychiatry, University Hospital Infanta Leonor, Madrid; 8 Psychiatry, University Hospital Complex of Santiago de Compostela, Santiago de Compostela; 9 Psychiatry, Hospital Complex of Segovia, Segovia; 10 Psychiatry, University Hospital Doctor Negrín, Las Palmas de Gran Canaria; 11 Psychiatry, University Hospital of Ribera, Alzira; 12 Psychiatry, University Hospital San Cecilio, Granada; 13 Psychiatry, Hospital Lluis Alcanyis, Xátiva; 14 Psychiatry, Advanced Neurology Center, Sevilla; 15 Psychiatry, University Hospital Complex of Ferrol, Ferrol; 16 University Hospital San Jorge, Huesca; 17 University Hospital of Donostia, San Sebastián, Spain

## Abstract

**Introduction:**

Major depressive disorder affects 280 million people globally and is a major cause of disability (OMS 2023). Treatment-resistant depression (TRD) is associated with poorer quality of life, greater functional impairment, reduced work performance, and higher healthcare use (Jaffe et al. BMC Psych 2019;19:11). Intranasal esketamine (IN-ESK) has demonstrated efficacy and safety in TRD (Papakostas et al. J Clin Psych 2020;81:19r12889). Functional recovery and work reintegration are increasingly recognized as essential treatment goals.

**Objectives:**

To assess clinical and functional outcomes of INK-ESK in patients with TRD treated in routine clinical practice.

**Methods:**

Retrospective, observational, multicenter study in 17 Spanish hospitals, approved by the local ethics committee. Subjects: patients ≥18 years, meeting DSM-5 criteria for MDD and TRD, treated with IN-ESK by their psychiatrist. Data: baseline sociodemographic and clinical characteristics, 12-month follow-up. Assessments: depressive severity (Montgomery-Åsberg-Depression-Rating-Scale-MADRS). Outcomes: clinical response (≥50% reduction in baseline MADRS), clinical remission (MADRS<7), and work reintegration. Time points: 4 weeks, 6 months, 12 months. Analysis: means, frequencies, chi-squared comparisons.

**Results:**

356 patients were included (mean age 52 years, 63% women, 38% on sick leave). Mean episode duration was 19 months; 49% lasted >1 year. Patients had failed a mean of four antidepressants (range 2–15); 35% remained non-responsive after five or more. Remission was achieved in 40% (27% ≤4 weeks) and response in 36%. Remission was higher in episodes <2 years (45% vs 25%, p=0.003) and tended to be higher in patients <30 years (53% vs 40%). Among 142 patients on sick leave, 49% achieved remission vs 35% not on leave (p=0.006), especially if leave ≤6 months (63% vs 42%, p=0.02). Return to work was 18% at 6 months and 22% at 12 months; early return more frequent with leave ≤6 months (36% vs 14%, p=0.002). Patients <30 years also returned to work more often than older patients (38% vs 19% at 6 months; 71% vs 27% at 12 months, ns).

**Conclusions:**

IN-ESK led to 40% remission and 36% response rates in TDR patients. Shorter episode duration and earlier treatment initiation were associated with higher remission and functional recovery. Patients on sick leave showed higher remission rates, particularly when leave was brief. Work reintegration was modest but more likely with shorter sick leave. Younger age may predict better outcome. Further studies are needed to confirm these results.

**Disclosure of Interest:**

A. De Santiago-Diaz Grant / Research support from: Johnson&Johnson: grant for 34th EPA congress and funding for clinical studies., Speakers bureau of: Johnson& Johnson, M. Madrazo-Maza Speakers bureau of: Johnson&Johnson, C. Pavón-Navajas: None Declared, A. Pérez-Poza: None Declared, J. Mesones-Peral Speakers bureau of: Johnson&Johnson, E. Sáez-Fuentes Speakers bureau of: Johnson&Johnson, F. Mora-Mínguez Speakers bureau of: Johnson&Johnson, M. Arrojo-Romero Speakers bureau of: Johnson&Johnson, A. Miranda-Sivelo : None Declared, F. Rodríguez-Batista: None Declared, V. Roselló-Molina Speakers bureau of: Johnson&Johnson, L. Gutiérrez-Rojas Speakers bureau of: Johnson&Johnson, V. Tordera-Tordera: None Declared, I. Domingos-Chaves: None Declared, D. Núñez-Arias Speakers bureau of: Johnson&Johnson, L. Montes Reula: None Declared, S. Arostegui-Uranga Speakers bureau of: Johnson&Johnson

